# Development of a Microbioreactor for *Bacillus subtilis* Biofilm Cultivation

**DOI:** 10.3390/mi15081037

**Published:** 2024-08-15

**Authors:** Mojca Seručnik, Iztok Dogsa, Lan Julij Zadravec, Ines Mandic-Mulec, Polona Žnidaršič-Plazl

**Affiliations:** 1 Faculty of Chemistry and Chemical Technology, University of Ljubljana, Večna pot 113, SI-1000 Ljubljana, Slovenia; mojca.serucnik@fkkt.uni-lj.si (M.S.); zadravec@fkit.unizg.hr (L.J.Z.); 2 Biotechnical Faculty, University of Ljubljana, Večna pot 111, SI-1000 Ljubljana, Slovenia; iztok.dogsa@bf.uni-lj.si (I.D.); ines.mandicmulec@bf.uni-lj.si (I.M.-M.); 3 Chair of Micro Process Engineering and Technology—COMPETE, University of Ljubljana, Večna pot 113, SI-1000 Ljubljana, Slovenia

**Keywords:** *Bacillus subtilis*, biofilm, microbioreactor, continuous cultivation

## Abstract

To improve our understanding of *Bacillus subtilis* growth and biofilm formation under different environmental conditions, two versions of a microfluidic reactor with two channels separated by a polydimethylsiloxane (PDMS) membrane were developed. The gas phase was introduced into the channel above the membrane, and oxygen transfer from the gas phase through the membrane was assessed by measuring the dissolved oxygen concentration in the liquid phase using a miniaturized optical sensor and oxygen-sensitive nanoparticles. *B. subtilis* biofilm formation was monitored in the growth channels of the microbioreactors, which were designed in two shapes: one with circular extensions and one without. The volumes of these microbioreactors were (17 ± 4) μL for the reactors without extensions and (28 ± 4) μL for those with extensions. The effect of microbioreactor geometry and aeration on *B. subtilis* biofilm growth was evaluated by digital image analysis. In both microbioreactor geometries, stable *B. subtilis* biofilm formation was achieved after 72 h of incubation at a growth medium flow rate of 1 μL/min. The amount of oxygen significantly influenced biofilm formation. When the culture was cultivated with a continuous air supply, biofilm surface coverage and biomass concentration were higher than in cultivations without aeration or with a 100% oxygen supply. The channel geometry with circular extensions did not lead to a higher total biomass in the microbioreactor compared to the geometry without extensions.

## 1. Introduction

Dramatic changes in biology and biotechnology (e.g., molecular microbiology, genomics, and systems biology) have spurred on the search for and the development of novel tools for the cultivation of microbial, plant, or animal cells, either for in vitro analytics, for the development of new biological products, or for the development and optimization of bioprocesses. The long tradition of using Petri dishes and shake flasks changed in the 1980s with the introduction of microtiter plates for cell cultivation, which allow a higher throughput, but have the major disadvantage that they offer only weak control over the process conditions and are not comparable to large-scale reactors [1].

The last decade has seen more radical changes as advances in microfabrication technology have contributed to the development of microfluidic systems that provide unique, more physiologically relevant environments for culturing cells, including the ability to precisely control the microenvironment at a scale relevant to microorganisms. The small size of microflow devices enables efficient heat and mass transfer, where flow is typically laminar and interfacial forces dominate over gravitational forces. Miniaturized devices offer the simplicity of control and flexibility of large bioreactors, the possibility for online monitoring of process conditions, automation, and operation in continuous mode. They also allow for high-throughput experiments under well-controlled conditions that enable real-time acquisition and better interpretation of experimental data, high-throughput screening of microorganisms and isolation of strains with improved production capabilities, and in operando testing of different process conditions, which is crucial for successful transfer to the production scale [1,2,3]. They are also ideal for analyzing minute amounts of material, for medical diagnostics, and for bioassays [4].

*Bacillus subtilis* is a Gram-positive bacterium that has attracted intense scientific interest due to its genetic amenability and interesting life cycle (spore development). It serves to answer many novel biological questions related to biofilms, such as, for example, spatial segregation duo to kin discrimination [5,6] and social evolution [7], and has considerable industrial importance as a producer of a variety of high-value biomolecules, e.g., enzymes, surfactants, and vitamins. Among the latter, vitamin B2 (riboflavin) is produced by genetically engineered *B. subtilis* in industrial quantities of over 2000 tons annually. This vitamin is required by all cells to assemble diverse flavoproteins, containing flavin mononucleotide (FMN) and flavin adenine dinucleotide (FAD), which catalyze many essential oxidation–reduction reactions [8]. Therefore, the intense interest from the industry in improving production processes drives the scientific interest in this microorganism.

Conventional engineering of *B. subtilis* strains to obtain strains with a higher productivity of target compounds, which requires parallel cultivation and further screening of a large number of candidates in Falcon tubes or microtiter plate formats, severely slows process development and increases its costs. In addition, the significantly different cultivation conditions compared to industrial bioreactors, e.g., regarding shear forces and oxygen transfer, represent a major drawback of these approaches, which might be diminished by using microfluidic devices [9]. For example, the dissolved oxygen concentration is one of the key parameters in riboflavin biosynthesis. Since oxygen can be efficiently introduced into microflow devices without altering the shear forces, e.g., by using permeable membranes or by introducing the gas phase into the microchannel [9], this opens up a great opportunity for detailed studies on the effect of oxygen on biofilm growth and metabolism.

Biofilms are multicellular communities, encased in self-produced extracellular polymeric substances (EPSs) that glue cells together and offer protection against hostile environments representing a dominant form of microbial life [10]. Biofilm formation is a stepwise process involving attachment, biofilm maturation, and dispersion stages [11,12]. Physiological states of biofilm-associated cells are highly regulated and often dependent on cell–cell signaling, also termed quorum sensing (QS), which controls in a density-dependent manner the EPS synthesis [13] and a variety of responses that may be of importance in biotechnological applications [14,15].

The bacterium *B. subtilis* has proved to be a good non-pathogenic model organism for the study of biofilm formation [13,16], as it forms highly robust multicellular structures that can be studied on abiotic and biotic surfaces, at the liquid–air interface [5,17], and even as biofilms submerged in liquid [18,19]. However, studies addressing *B. subtilis* biofilm development in microfluidic devices are still rare [20,21].

The aim of this study was to cultivate *B. subtilis* biofilms in a continuously operated microbioreactor to enable comprehensive investigations of biofilm growth under controlled conditions. Achieving this goal necessitated the development of a microfluidic device that allows aeration. The effects of different microbioreactor geometries and environmental conditions, i.e., flow rate and aeration, on the cultivation of *B. subtilis* biofilm were systematically investigated.

## 2. Materials and Methods

### 2.1. Microorganism

*Bacillus subtilis* VKPM B2216 was donated from the culture collection of Acies Bio d.o.o. (Ljubljana, Slovenia).

### 2.2. B. subtilis Growth Media

Complete growth medium (CGM) consisted of 16 g/L tryptone, 10 g/L yeast extract (both Biolife, Milano, Italy), and 5 g/L NaCl (Merck, Billerica, MA, USA) (pH 6.8).

### 2.3. B. subtilis Cultivation in Shaken Cultures

We transferred 50 µL of the *B. subtilis* frozen stock culture with 10% glycerol to 50 mL of CGM in a baffled Erlenmeyer flask, which was placed in an incubator at 36 °C and shaken at 220 min^−1^ for 20 h. To prepare the inoculum for the microreactor, 5 mL from the overnight culture was transferred to 50 mL of the fresh CGM and placed in an incubator at 36 °C for another 4 h. The culture was checked for OD_600_ using a Shimadzu UV 2600 spectrophotometer (Shimadzu, Kyoto, Japan).

### 2.4. Microbioreactor Components

A microfluidic device was constructed by assembling multiple layers to form microchannels, following a similar concept to our previous research [22,23]. On the top of the microfluidic device was a 25 × 60 × 15 mm^3^ polymethyl methacrylate (PMMA) block with two inlet and two outlet ports for connection to perfluoroalkoxy (PFA) tubes (1.59 mm OD, 0.5 mm ID) via male mini-luer fluid connectors (Microfluidics ChipShop, Jena, Germany). The channels for the gas and liquid phases, each with different geometries (see Section 3.1), were cut from two 143 μm thick, double-sided adhesive polypropylene films—Arseal 90,880 (Adhesives Research, Limerick, Ireland)—using a Cameo 4 precision cutting machine (Silhouette America, Lindon, UT, USA). A 24 × 60 mm^2^ # 1.5 cover glass (Menzel Gläser, Braunschweig, Germany) served as the base plate of the microbioreactor.

To prepare a polydimethylsiloxane (PDMS) membrane, the silicone elastomer base and the curing agent (both from the Sylgard 184 Silicone Elastomer Kit, Dow Chemical Company, Midland, MI, USA) were mixed in a mass ratio of 9:1. The resulting elastomer was evenly applied to a Teflon film (Dastaflon d.o.o., Medvode, Slovenia) using a stainless steel thin-film applicator (Erichsen, Hemer, Germany) to form a 200 µm thick layer, which was cured at 90 °C for 20 h. The final depth of the resulting PDMS membrane was assessed using a Leica DM IL LED inverted microscope (Leica, Wetzlar, Germany) equipped with a Leica MC 190 HD digital camera. Bright-field microscopic images were acquired using an N PLAN L 40×/NA 0.55 objective, and Leica Application Suite image acquisition software (version 3.4.0) facilitated the image acquisition process. The membrane was positioned laterally, and its depth was measured at several points, ensuring that at least three measurements were taken at each membrane to obtain an average value and the corresponding statistical deviation.

### 2.5. Determination of the Microbioreactor Volume

A precise high-pressure syringe pump (Pump 33 DDS, Harvard apparatus, Holliston, MA, USA) was used to determine the volume of the growth channel in the microbioreactor. The microbioreactor was assembled as described in Section 3.1, and a food dye solution was used for better visualization. The solution was first pumped to the beginning of the channel, and the pump volume was set to zero. Then, the solution was pumped into the channel at a flow rate of 4 µL/min, and the volume was recorded when the solution reached the end of the channel.

The procedure was repeated three more times with the same device. The first measurement was always smaller, but from the second measurement onward, the volume stabilized, indicating that it corresponded better to the actual conditions. The volume determination was carried out for three microbioreactors with rectangular growth channels and for five microbioreactors with circular extensions, and the average values were calculated with standard deviations.

### 2.6. Evaluation of Oxygen Transfer within the Microbioreactor

To assess the oxygen transfer from the gas phase through the PDMS membrane into the liquid phase inside the microbioreactor, dynamic experiments were performed with nitrogen and oxygen gases at room temperature. The concentration of dissolved oxygen in the liquid phase was monitored using ultrapure water with dissolved OXNANO oxygen detection nanoparticles (Pyroscience, Aachen, Germany) [24]. A solution containing 10 mg of nanoparticles in 3 mL of ultrapure water was sonicated for 10 min before being pumped into the microbioreactor using a syringe pump (Essi tech d.o.o., Ljubljana, Slovenia). The reaction of the nanoparticles was measured using a Piccolo_2_ detector (Pyroscience, Aachen, Germany), and the data were recorded using Pyro Oxygen Logger software [25]. The detector was positioned under the central point of the glass coverslip. After recording a blank measurement with the reactor empty, the channel above the coverslip (under the PDMS membrane) was filled with the indicator solution, and liquid and gas flows were introduced in opposite directions. To ensure a controlled gas flow into the microbioreactor, the electronically controlled valve EL-FLOW Prestige (Bronkhorst, Ruurlo, The Netherlands) was used.

To calibrate the detector, nitrogen was first introduced into the gas-phase channel at a flow rate of 2 mL/min until the detector readings stabilized in the liquid phase, indicating a dissolved oxygen concentration of 0 mg/L. Subsequently, 100% oxygen was introduced into the channel for the gas phase until the detector readings stabilized, indicating a saturated dissolved oxygen concentration of 40 mg/L.

After calibration, oxygen transfer tests were performed at liquid flow rates of 5 and 10 µL/min while maintaining a constant gas flow rate of 2 mL/min. The gas supplied to the reactor was switched from 100% nitrogen to 100% oxygen in repeated cycles. The oxygen transfer was determined from the linear part of the curve, in which the dissolved oxygen concentration was plotted against time. The average value of the data obtained from three experiments and the corresponding standard deviation were calculated.

### 2.7. Inoculation and Cultivation of B. subtilis in the Microbioreactor

The liquid-phase channel of the microbioreactor was sterilized by pumping 70% ethanol through it with a syringe pump. After sterilization, the bacterial culture was injected into the liquid-phase channel to displace any residual ethanol. A sufficient amount of the bacterial culture suspension with a concentration of 10^9^ cells/mL was injected into the microbioreactor, so that the liquid-phase channel was completely filled and a drop of liquid formed at the end of the outlet tube. The entire system was then allowed to stand for 45 min, with continuous monitoring to ensure that the droplet at the outlet tube did not evaporate. If evaporation occurred, a small amount of the liquid culture was injected into the channel to restore the droplet on the outlet tube. At the end of the 45 min period, the syringe with the inoculum was replaced with a syringe with the growth medium, as described in Section 3.

The growth medium was then pumped through the liquid-phase channel of the microbioreactor at flow rates of 0.3 or 1 µL/min. The microbioreactor, which was placed on a heated plate (IKA, Staufen, Germany), was set to a temperature of 36 °C. Depending on the experimental parameters, the inlet and outlet ports for the gas supply in the upper channel were either covered with tape to prevent air from entering, or a tube for the gas supply was inserted into the inlet, through which either air or 100% oxygen was supplied at a flow rate of 1 mL/min. The gas phase was passed through a cotton filter and regulated by an electronically controlled valve (Bronkhorst, Ruurlo, The Netherlands).

For each incubation condition, two to three independent replicates were performed either simultaneously in parallel microreactors or sequentially.

### 2.8. Analysis of Biofilm Coverage, Statistics, and Cell Morphology in the Biofilm

To monitor biofilm coverage within the growth channel, photographs of the microbioreactor were taken at specific time intervals. For each time point, two types of images were taken—one for better visualization of the grown biofilm (black background and light biofilm), and one for quantitative analysis of the biofilm coverage and its optical density (white background, dark biofilm). In the first case, the bioreactor, which is translucent, was placed on a black surface and illuminated with ambient light. In the second case, the bioreactor was placed on a uniformly illuminated LCD screen and shielded from ambient light. To obtain images with constant camera settings, which was particularly important for quantifying the optical density (*OD*) of the biofilm, a Canon EOS 600D camera (Canon, Tokyo, Japan) was operated in manual mode (ISO 100, f/25, 1/10 s). A raw CR2 image format with 18 megapixels was used for image quantification [26].

In the first step, the individual color images were broken down into three grayscale images, each representing a specific color channel (blue, red, and green). The red color channel was selected for further processing due to its optimal contrast. The outline of the microbioreactor channels was manually traced on a selected red channel image, followed by a conversion of the grayscale pixel intensities to *OD* according to Equation (1):

*OD* = *log* (*I*/*I*_0_) (1)

where *I*_0_ is the intensity of the incident light, i.e., the intensity of the grayscale pixels in the image on the surface of the LCD screen, and *I* is the intensity of the incident light through the biofilm, i.e., the intensity of the grayscale pixels in the image of the upper part of the bioreactor channel. The final *OD* reported in the results was determined by subtracting the optical density of the empty channel (*OD*_0_), i.e., the channel filled only with sterile growth medium, from the *OD* of the channel filled with biofilm. The biofilm-covered surface was defined as the channel surface with an *OD* ≥ *OD*_0_ + *sd*_0_, where *sd*_0_ is the standard deviation of the *OD*_0_ in the same channel calculated from the variability in the grayscale pixel intensity of the channel. The surface coverage reported in the results was then calculated as the ratio between the surface area covered by the biofilm and the total surface area of the microchannel. The surface coverage of the empty channel, which was only filled with sterile growth medium, was subtracted from the surface coverage of the biofilm-filled channel. The experimental errors of the individual time points were calculated as standard deviations determined from the *OD* and the surface coverages of two consecutive images from the same microbioreactors.

A two-tailed, unpaired Student’s *t*-test with unequal variances was used to calculate the statistical significance of the data sets. A *p*-value of less than 0.05 was considered statistically significant. Only independent replicates of the experiments (i.e., different chips) were considered in the *t*-test. For the comparison of biomasses in the microchannels under different conditions calculated based on *OD* and microchannel volume, the error propagation formula was first used to derive the standard deviation of biomasses and then a *t*-test was performed. This analytical approach provided a quantitative measure of the biofilm coverage and biomass accumulation over time, offering valuable insights into the progression and dynamics of biofilm formation in the microbioreactor.

For continuous observation of the biofilm microstructure at the microscopic level without disrupting the flow of the growth medium, the microbioreactor was temporarily moved from the incubation plate to the stage of an inverted microscope with a digital camera (see Section 2.4). Manual adjustments to the placement of the microbioreactor on the microscope stage enabled a comprehensive observation of the biofilm in all areas of the growth channel. This microscopic analysis provided detailed insights into the morphology and spatial distribution of the biofilm during the different growth stages.

## 3. Results and Discussion

### 3.1. Development of a Microbioreactor

**Figure 1:**
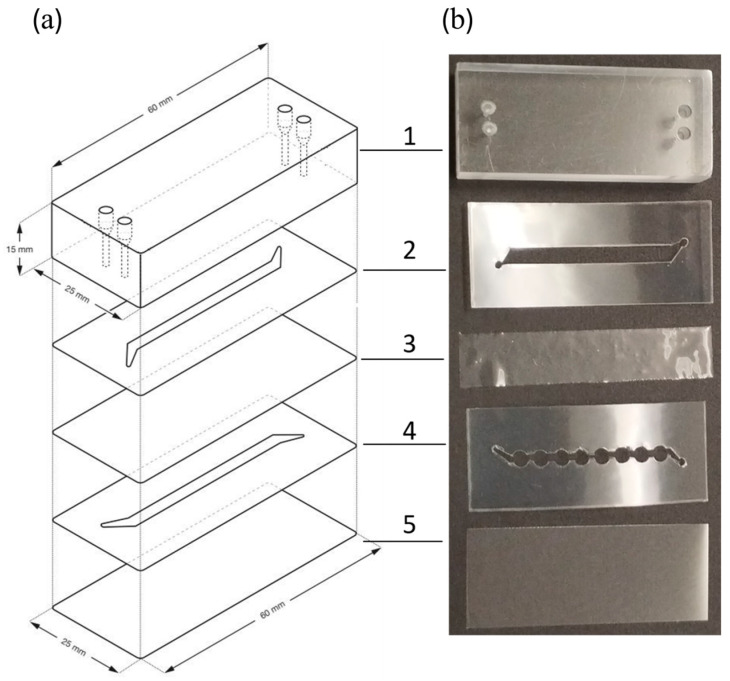
Structure of the microbioreactor, consisting of 1—PMMA plate with holes for the liquid and gas supply, 2—double-sided adhesive polypropylene foil with a carved channel for the gas supply, thickness 143 μm, 3—PDMS membrane, thickness 108.5 ± 0.9 μm, 4—double-sided adhesive polypropylene foil with a carved channel for the bacterial culture, thickness 143 μm, 5—glass coverslip. (**a**) Schematic presentation of a microbioreactor with a rectangular growth channel; (**b**) photos of the components of the microbioreactor with circular extensions of the rectangular growth channel.

During the development of the microbioreactor, two variants with different channel geometries were tested for bacterial biofilm cultivation. The cover glass was selected as the base of the microbioreactor, which also determined the dimensions of the subsequent layers. As illustrated in Figure 1a, the microbioreactors were assembled by fixing a carved polypropylene layer onto the PMMA block, which contained holes for the tubing connections. This arrangement ensured that the inlet and outlet for the upper channel aligned with the corresponding holes in the PMMA block. A PDMS membrane and another carved polypropylene sheet were then added, with the latter positioned so that the inlet and outlet ports of the lower channel aligned with a second pair of holes in the PMMA block. The foil and membrane were perforated to allow liquid to flow in and out of the lower channel through the holes in the PMMA block. Finally, the lower channel was covered with a glass coverslip. The latter was selected as the bottom layer to facilitate observation of the lower channel with an inverted microscope. For the same reason, all inlet and outlet openings were drilled in the upper PMMA block. The double-sided adhesive polypropylene foil was used to construct the channel scaffold and hold the entire reactor assembly together. The PMMA block provided the structural strength of the reactor and allowed the inlet and outlet tubes to be attached. In a final step, all components had to be pressed onto the flat surface with sufficient force to ensure a firm connection. The reactors were assembled by hand while the adhesive foils were cut with a Cameo cutter to ensure uniform channel dimensions. The assemblies were tested for leaks, which was a problem in approximately 20% of cases.

**Figure 2:**
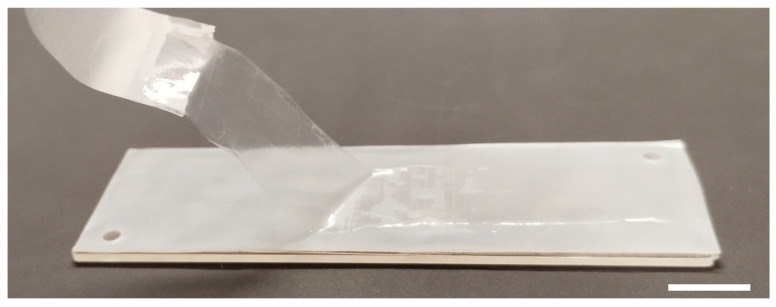
Removal of the transparent PDMS membrane from the Teflon foil after incubation in the oven. The membrane was used to transfer oxygen from the gas to the liquid phase within the microbioreactor. To facilitate detachment from the support, the front edge of the membrane was placed on adhesive tape. The scale bar corresponds to 10 mm.

The self-produced PDMS membrane used for the oxygen transfer from the gas to the liquid phase in the microbioreactor is shown in Figure 2. Microscopic assessment of its final depth after incubation at 90 °C for 20 h, as described in Section 2.4, revealed an average value of 108.5 ± 0.9 μm.

Two separate channels were cut out of the polypropylene layers to facilitate the supply of gasses and liquids. In both versions of microbioreactors, the upper microchannel dedicated to the gas phase and the lower microchannel for the growth of *B. subtilis* were made in a rectangular shape, creating a free space for the gas. The primary channels were connected to the inlet and outlet ports via 5 mm long and 1 mm wide side channels arranged at an angle of 60° to the main channel, as shown in Figure 1a,b-2.

In the first version of the microbioreactor, the upper and the lower channels had the same dimensions of 37.5 mm length, 1.9 mm width, and 143 µm depth, but the side channels led in opposite directions to the inlet and outlet ports, as depicted in Figure 1a. Each microchannel had a single surface area of 75 mm^2^, resulting in a void volume of approximately 17 µL.

In the second version of the microbioreactor, shown in Figure 1b, the top microchannel for the gas phase had a length of 37.5 mm, a width of 3.9 mm, and a depth of 143 µm, while the growth channel was the same as in the first microreactor but had eight circular extensions, each with a diameter of 3.94 mm. This microbioreactor had a growth channel surface area of about 110 mm^2^ and a volume of about 28 µL.

The PFA tubing used for both gas and liquid supply was connected to the microbioreactor via male mini-luer fluid connectors that fit securely into the holes drilled in the PMMA block. This connection mechanism ensured a reliable and tight interface between the tubing and the microbioreactor and allowed controlled gas and liquid flows within the system.

### 3.2. Characterization of Oxygen Transfer Rate within the Microbioreactor

**Figure 3:**
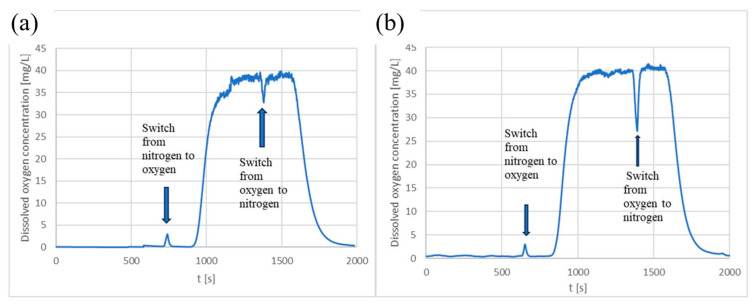
Concentration of dissolved oxygen in a liquid solution containing an indicator in the form of nanoparticles. The flow rate of the gas (starting with nitrogen, and further changes are indicated on the graph) was 2 mL/min, while liquid flow rates were (**a**) 5 µL/min and (**b**) 10 µL/min. Only one of the three replicates is shown, while their standard deviations are listed in Table 1.

After assembling the microbioreactor with a rectangular growth channel with PDMS membrane, shown in Figure 2, dynamic oxygen transfer tests were conducted to assess the efficiency of oxygen supply in the liquid phase. The liquid solution containing an oxygen indicator was pumped into the reactor in the opposite direction with nitrogen or oxygen through the corresponding channels shown in Figure 3. The dissolved oxygen concentration in the indicator solution was then measured as described in Section 2.6.

Since no microorganisms were present in this experiment, the oxygen accumulation was proportional to the transfer of oxygen from the gas phase through the PDMS membrane into the liquid solution containing an oxygen indicator in the form of nanoparticles. The dissolved oxygen in the liquid solution was measured using an optical detector placed under the central point of the lower glass slide. Figure 3 shows a portion of the data representing the dissolved oxygen concentration over time at two different liquid flow rates.

As demonstrated in Figure 3 and Table 1, the increase in dissolved oxygen concentration in the liquid phase was very fast and similar for both tested liquid flow rates, namely, 5 and 10 µL/min. This observation indicates that the membrane exhibits a high permeability to oxygen, implying that the microbioreactor is well suited for the cultivation of aerobic microorganisms.

### 3.3. Testing the Microbioreactor’s Ability to Support Biofilm Growth

The first experiments were performed in the microbioreactor illustrated in Figure 1a with a rectangular growth channel. After inoculation of the bacterial culture with 10^6^ *B. subtilis* cells obtained from shaken cultures (Section 2.3), the complete growth medium (CGM) outlined in Section 2.2 was introduced into the microbioreactor at a flow rate of 1 μL/min. At the same time, air or oxygen was introduced into the microchannel above the PDMS membrane at a flow rate of 1 mL/min in the opposite direction.

**Figure 4:**
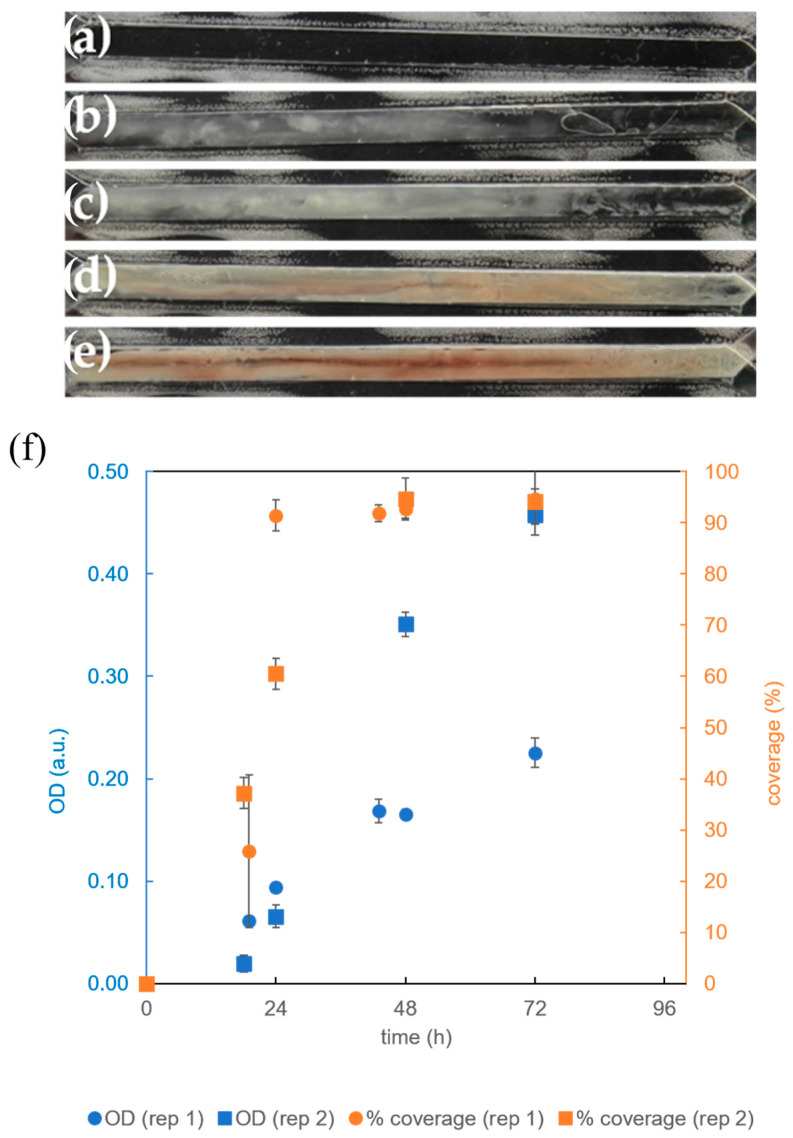
Cultivation of *B. subtilis* biofilm in a microreactor with a rectangular growth channel in CGM at 36 °C, an air flow rate of 1 mL/min, and a growth medium flow rate of 1 μL/min. (**a**) Growth channel filled with CGM; (**b**) biofilm after 18 h, (**c**) after 24 h, (**d**) after 48 h, and (**e**) after 72 h; (**f**) time dependence of two replicates (rep 1 and rep 2) of the surface coverage of the rectangular growth channel by the *B. subtilis* biofilm (orange) and time dependence of the optical density (*OD*) within the growth channel (blue) as a quantitative measure of biofilm coverage and biomass accumulation. Note that the white spots in figures (**a**–**e**) outside the channel are due to the adhesive between the film and the PDMS membrane.

In the first 24 h, most of the channel volume was taken up by the biofilm (Figure 4c,f). At the same time, the *OD* increased significantly compared to the initial *OD* in both replicates (*p* < 0.02). However, the final bacterial density in the biofilm increased 5- to 10-fold over the next two days, indicating that the bacteria first occupied the available surface area and then the entire volume. By the third day, the biofilm had expanded to cover most of the channel (Figure 4c), and by the sixth day, the channel was completely covered with biofilm (Figure 4d).

The final biomass in the channel can be estimated based on the bacterial density and channel volume. Previous studies have shown that the bacterial density at an *OD* = 1.0 a.u. and an optical path length of 10 mm is approximately 4 × 10^8^ *B. subtilis* cells/mL [27]. From the known volume of the channel and surface area, which were (17 ± 4) μL and (73 ± 1) mm^2^, respectively (Table 2), the average height of the channel could be calculated, which was (0.23 ± 0.05) mm. After 72 h of incubation, the *OD* was (0.3 ± 0.15) a.u. (Figure 4f), which corresponds to a bacterial density of (5 ± 3) × 10^9^ *B. subtilis* cells/mL and a total cell number of (9 ± 5) × 10^7^ *B. subtilis* cells per channel.

After 48 h of cultivation, a red dye was detected in the biofilm of *B. subtilis* (Figure 4d). This was probably a pulcherrimin pigment, which was formed by the reaction of the colorless pulcherrimic acid with Fe^3+^ ions in the yeast extract of the growth medium [28]. In previous studies, this pigment was only observed in *B. subtilis* bacteria growing in biofilms on a solid surface, and not in the biofilms growing in suspension. Pulcherrimin serves to reduce oxidative stress by inhibiting the formation of reactive oxygen species and protecting the spores from UV light [29,30,31].

**Figure 5:**
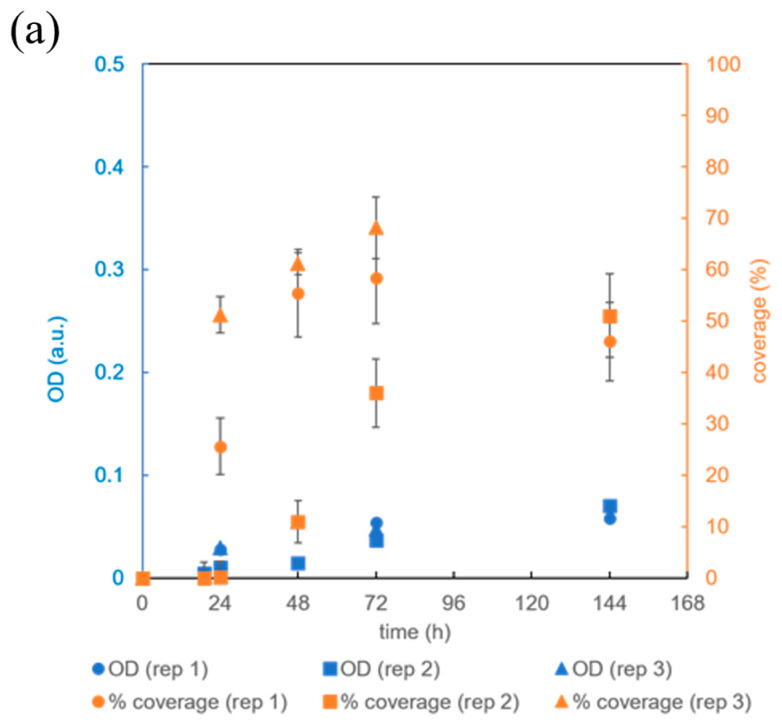

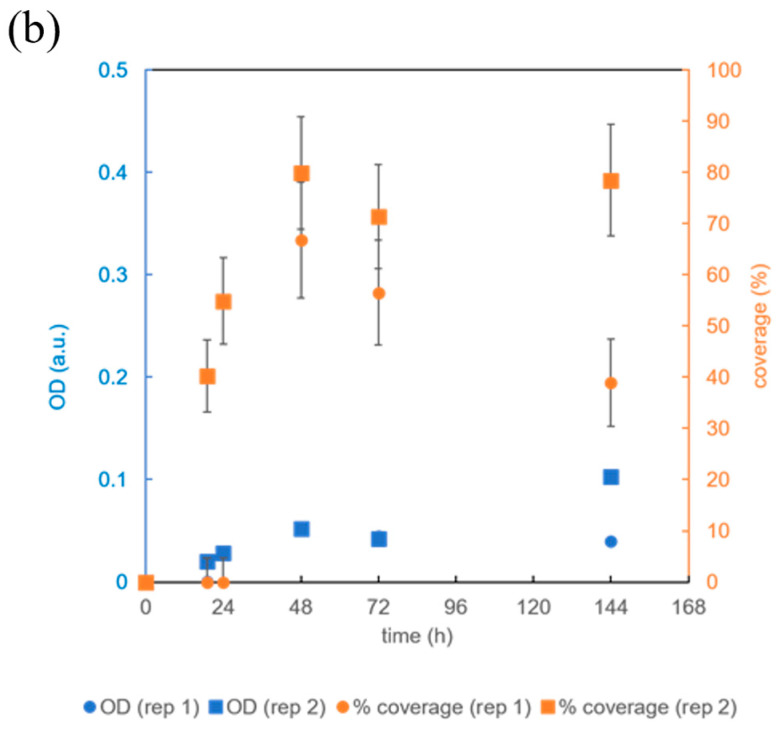
Time dependence of *B. subtillis* biofilm coverage of the rectangular growth channel surface (orange) and time dependence of optical density (OD) within the growth channel (blue) as a quantitative measure of biofilm coverage and biomass accumulation in a complete growth medium (**a**) at a flow rate of 1 μL/min and (**b**) at a flow rate of 0.3 μL/min at 36 °C. The flow rate of 100% oxygen was 1 mL/min in both cases. The replicate experiments are referred to as rep 1, rep 2, and rep 3.

To investigate the potential for inducing different biofilm growth in the designed microbioreactor, *B. subtilis* was exposed to significantly different growth conditions. The air was replaced by 100% oxygen, while at the same time, the flow rate of the growth medium was reduced to 0.3 µL/min; the results of experiments are shown in Figure 5. A comparison of Figure 4f with Figure 5a shows that supplying 100% oxygen instead of air significantly reduced surface coverage and *OD* (*p* < 0.05 at *t* = 72 h). In contrast, reducing the flow rate of the growth medium (Figure 5b) did not lead to significant changes in *OD* or surface coverage. However, the probability of unwanted bubble formation was lower at a higher flow rate. We also observed that the occasional washout of the biofilm did not occur at the higher growth medium flow rate.

These results demonstrate that our newly developed microbioreactor is well suited to biofilm growth and is sensitive to changing growth conditions. To gain further insights into biofilm growth in microbioreactors, we systematically varied the geometry of the growth channel and the individual growth conditions.

### 3.4. The Effect of Microbioreactor Geometry on B. subtilis Biofilm Cultivation

**Figure 6:**
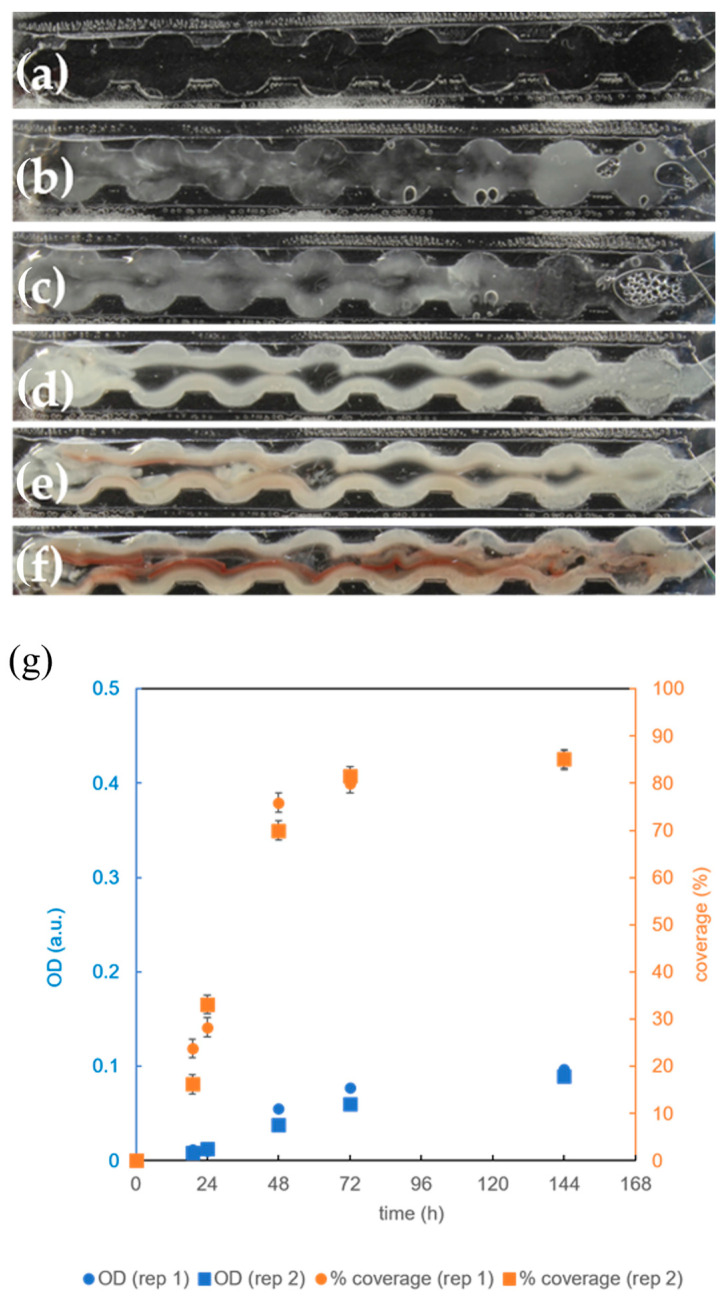
Cultivation of *B. subtilis* biofilm in a microreactor with a rectangular growth channel with circular extensions at 36 °C and with a complete growth medium (CGM) flow rate of 1 μL/min and an air flow rate of 1 mL/min: (**a**) growth channel filled with CGM; (**b**) biofilm after 19 h, (**c**) after 24 h, (**d**) after 48 h, (**e**) after 72 h, and (**f**) after 144 h; (**g**) time dependence of *B. subtillis* biofilm surface coverage of the growth channel (orange) and time dependence of the optical density (*OD*) within the growth channel (blue) as quantitative measure of the biofilm coverage and biomass accumulation. The replicate experiments are referred to as rep 1 and rep 2.

To examine the effect of channel geometry on bacterial biofilm formation and to mitigate biofilm detachment, a new growth microchannel was designed with eight circular expansions along its length (Figure 1b-6 and Table 2). These expansions created areas in the microbioreactor where liquid flow was slower, presumably enabling better biofilm retention [32,33]. This was indeed the case, as we observed relatively strong biofilm growth on the edges of the extensions (Figure 6).

However, after 24 h of incubation (Figure 6c), the biofilm showed a gap in the center, where the flow rate of the growth medium was highest. This phenomenon can be attributed to the movement of bacteria to the edges of the channel under the influence of shear stress caused by the flow of the growth medium [21,34]. Contrary to our expectations, the *OD* and surface coverage were not greater in the microchannels with circular extensions (Figure 6g) than in those without them (Figure 4f). Even when accounting for the larger volume (Table 2) of the microchannels with circular extensions, the estimated average biomass was higher in the microchannels without circular extensions [(9 ± 5) × 10^7^ cells vs. (4 ± 1) × 10^7^ cells], although the difference was not statistically significant (*p* = 0.30).

The synthesis of a red pigment responsible for attenuating oxidative stress in the biofilm of *B. subtilis* was also observed only after 72 h (Figure 6e), while it was present after 48 h in the case of a rectangular growth channel without circular extensions (Figure 4d). Taken together, these results indicate that the growth conditions for biofilm formation in the microchannels with circular extensions are different and possibly less favorable than in microchannels without circular extensions. One possible explanation for this is that due to the geometry of the circular extensions, most of the nutrients flow in a narrow stream through the center of the channel and the bacteria, which are pushed to the edges of the channel, receive the nutrients mainly by diffusion.

### 3.5. The Effect of Aeration on B. subtilis Biofilm Cultivation in the Microbioreactor with Circular Extensions

**Figure 7:**
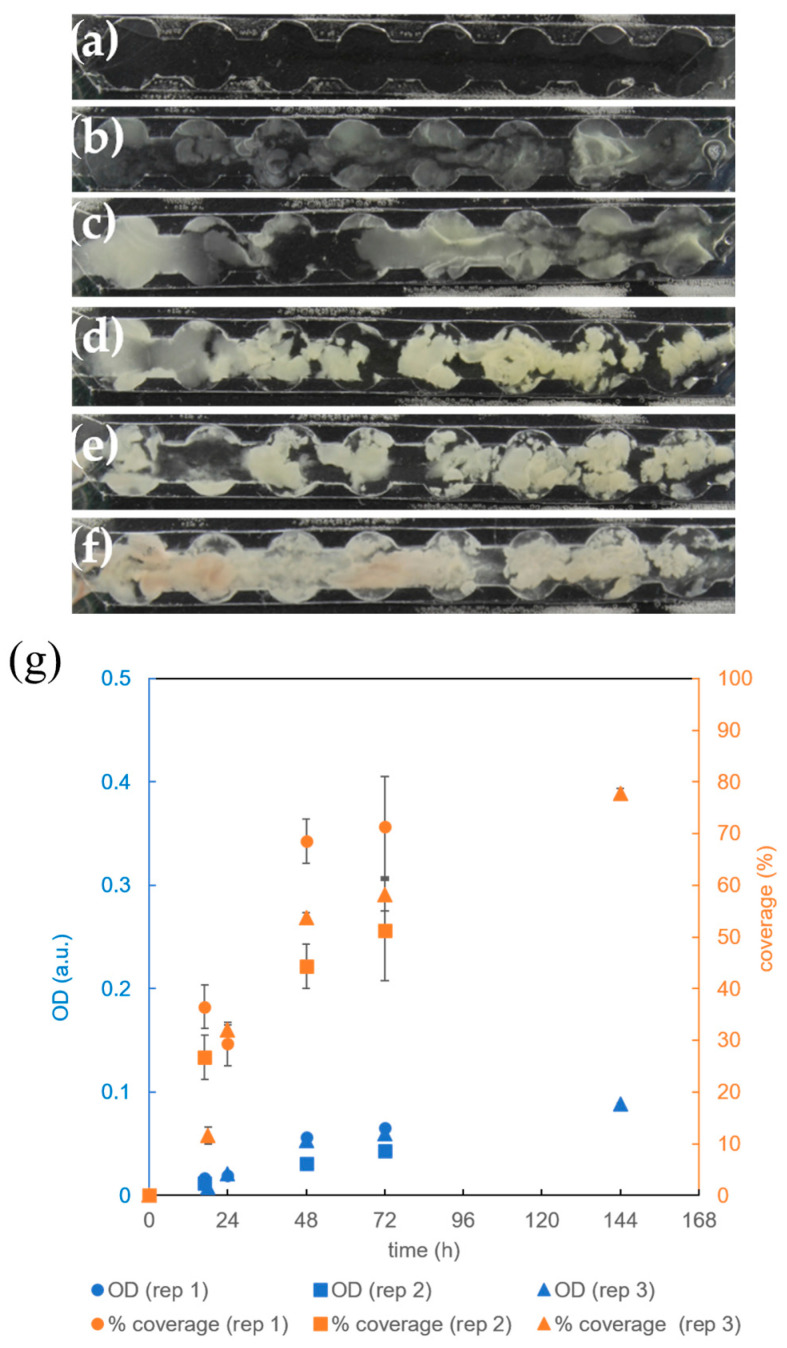
Cultivation of *B. subtilis* biofilm in a microreactor with a rectangular growth channel with circular extensions at 36 °C and in a complete growth medium (CGM) introduced at a flow rate of 1 μL/min and 100% O_2_ supplied with a flow rate of 1 mL/min. (**a**) Growth channel filled with CGM; (**b**) biofilm after 17 h, (**c**) 24 h, (**d**) 48 h, (**e**) 72 h, and (**f**) 144 h; (**g**) time dependence of *B. subtillis* biofilm surface coverage of the growth channel (orange) and time dependence of the optical density (*OD*) within the growth channel (blue) as quantitative measure of the biofilm coverage and biomass accumulation. The replicate experiments are referred to as rep 1 and rep 2.

Since it was shown that the supply of 100% oxygen instead of air significantly reduces the surface coverage in the microreactor without circular extensions (Section 3.3), the effect of aeration on *B. subtilis* biofilm cultivation was also tested for the microchannels with circular extensions. Indeed, we observed that the biofilm had difficulty covering the entire channel in the presence of pure oxygen (Figure 7a–f), and the distribution of bacteria was different from that in the presence of air supply (Figure 6a–f). While biofilm growth was considerable, adherence to surfaces was weaker, resulting in larger biofilm aggregates covering the center of the channel (Figure 7a–f). These aggregates were sensitive to the flow of the growth medium and were regularly moved in the direction of flow, as a comparison of Figure 7b–d shows.

The analysis suggests that, similar to the case of microchannels without circular extensions, the introduction of 100% oxygen slightly decreases the surface coverage after 144 h of cultivation (85% in Figure 6g vs. 78% in Figure 7g), although the difference was not statistically significant (*p* = 0.07). Similarly, the final *OD* of biofilms cultivated under 100% oxygen was comparable to the conditions under air supply [(0.1) a.u. in Figure 6g vs. (0.09) a.u. in Figure 7g], which was also confirmed by checking the statistical insignificance (*p* = 0.30). It can be concluded that the differences between the supply of air and pure oxygen are smaller in microchannels with circular extensions than in those without. This can be explained by generally weaker growth in microchannels with circular extensions (Figure 6g) compared to the channels without circular extensions (Figure 4f) in the presence of air.

**Figure 8:**
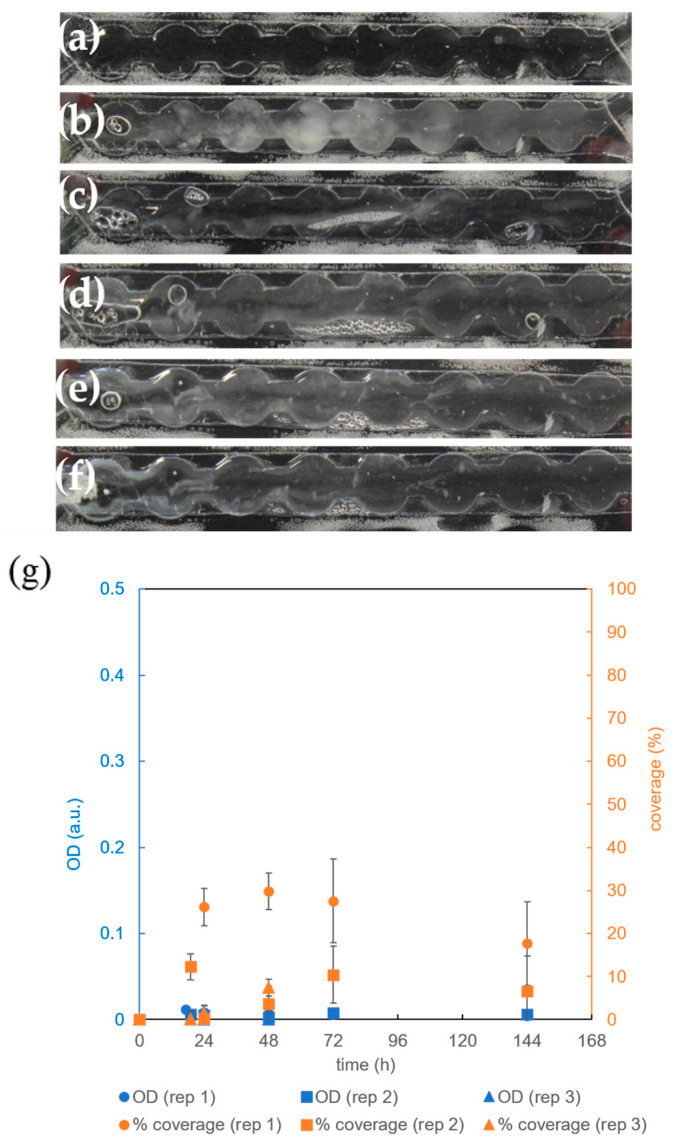
Cultivation of *B. subtilis* biofilm in a microreactor with a rectangular growth channel with circular extensions in CGM at 36 °C and without aeration: (**a**) growth channel filled with CGM; (**b**) biofilm after 19 h, (**c**) 24 h, (**d**) 48 h, (**e**) 72 h, and (**f**) 144 h; (**g**) time dependence of *B. subtillis* biofilm surface coverage of the growth channel (orange) and time dependence of the optical density (OD) within the growth channel (blue) as quantitative measure of the biofilm coverage and biomass accumulation. The replicate experiments are referred to as rep 1 and rep 2.

Further investigations focused on cultivation without aeration, in which the cells are solely dependent on the oxygen dissolved in the entering growth medium. As evident from Figure 8, biofilm growth was significantly weaker compared to growth with air or a 100% oxygen supply (Figure 6 and Figure 7). The total biomass in the microchannel after 72 h determined from the *OD* was ≤4 × 10^6^ cells (*p* < 0.009), which is about 10 times lower than with air supply (Figure 6). This reduced *OD* was accompanied by reduced surface coverage, reaching only (19 ± 12)% after 72 h, significantly less than with air supply at the same cultivation time [(81 ± 1)% at *p* < 0.02].

The pronounced effect of oxygen on biofilm growth in microbioreactors is consistent with the phenotypic properties of *B. subtilis*. This bacterium, often categorized as an aerobe, shows the ability to grow anaerobically only in the presence of nitrate, which can be used as an alternative electron acceptor, allowing *B. subtilis* to be more accurately described as a facultative anaerobe [35,36]. In the absence of nitrate in CGM, growth of *B. subtilis* in microchannels was dependent on oxygen. In low-oxygen environments, most *B. subtilis* cells tend to migrate to the surface and form biofilms (pellicles) at the air–liquid interface [17]. Although part of the biomass remains in the liquid phase and also accumulates at the bottom of the well, it is subjected to faster decay than the biomass at the surface [17]. The transition between the liquid–solid and liquid–air interfaces is influenced by the concentration of dissolved oxygen [18]. *B. subtilis* has an oxygen sensor that can modulate its chemotaxis [37]. Some studies suggest that oxygen promotes the adhesion of bacteria to the surface. Conversely, oxygen deficiency leads to poor cell adhesion and often serves as a signal for biofilm detachment, with the oxygen concentration strongly influencing biofilm formation [31,32]. This could explain the poor biofilm formation under non-aerated conditions. On the other hand, our results indicate that too much oxygen can also hinder biofilm formation in microbioreactors. This can be explained by the fact that increased oxygen concentrations generally lead to increased production of reactive oxygen species (superoxide and H_2_O_2_) from the respiratory chain, which affect metalloenzymes and DNA, which in turn can impair growth [38]. This is consistent with the reduced biofilm growth rate observed in our study (Figure 4f and Figure 5a). In addition, recent studies in shaken cultures have shown that an excessive oxygen concentration inhibits the production of ComX, a signaling molecule responsible for quorum sensing in *B. subtilis* [27]. ComX plays a role in regulating the expression of genes responsible for the synthesis of exopolymers [39,40]. This is important because exopolymers serve as adhesive components that facilitate the transition of planktonic cells into biofilms, and this could be an explanation for the reduced biofilm mass under the condition of 100% oxygen supply [13,41].

### 3.6. The Microscopic Properties of B. subtilis Biofilm Cultivated in a Microbioreactor

**Figure 9:**
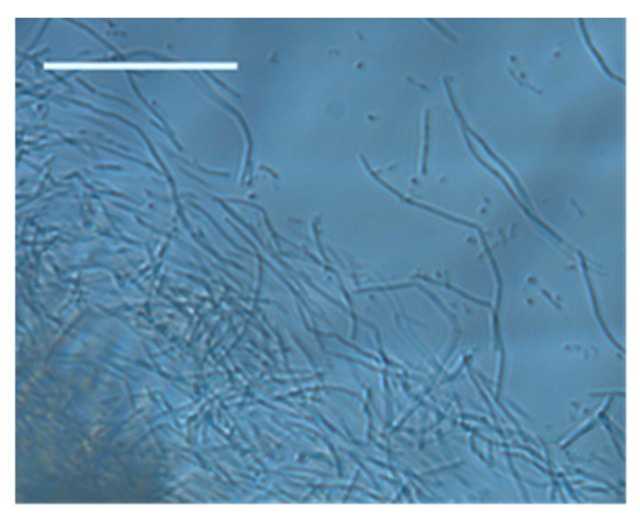
Phenotypic heterogeneity of *B. subtilis* cells in biofilm: long settler cells and short explorer cells after 72 h cultivation in a microbioreactor with circular expansions in complete growth medium introduced at a flow rate of 1 μL/min and an air flow rate of 2 mL/min. The scale bar represents 50 µm.

To demonstrate and confirm the suitability of the constructed microbioreactors for microscopic observation, the microbioreactors were also monitored by microscopic analysis. One phenomenon that was repeatedly observed in our experiments was the formation of two morphologically distinct, stable cell subpopulations in the biofilm (Figure 9), which is referred to as phenotypic heterogeneity [42,43]. One subpopulation consisted of “settlers” that formed long and immobile chains, which attached to the surface of the channel; another subpopulation, termed “explorers”, existed in the form of short, highly motile rods, which moved between the attached cells and floated in the medium flowing through the channel, consistent with the usual behavior of *B. subtilis* cultures [13,17].

Most bacteria were integrated into the biofilm on the glass surface, and some longer bacteria were present on the PDMS membrane. Adherence to both hydrophilic and hydrophobic surfaces can be explained by the fact that *B. subtilis* is able to produce different types of EPSs [10,13,17,44], some of which have properties that allow them to adhere to hydrophilic surfaces, while others can be bound to hydrophobic surfaces. For example, the protein BslA is known to form a hydrophobic biofilm surface in *B. subtilis* [45] and could thus facilitate the attachment to hydrophobic surfaces. On the other hand, γ-polyglutamic acid (PGA) is a hydrophilic polymer produced by *B. subtilis* that facilitates the attachment to hydrophilic plant root surfaces [46] and could be involved in adhesion to other hydrophilic surfaces.

**Figure 10:**
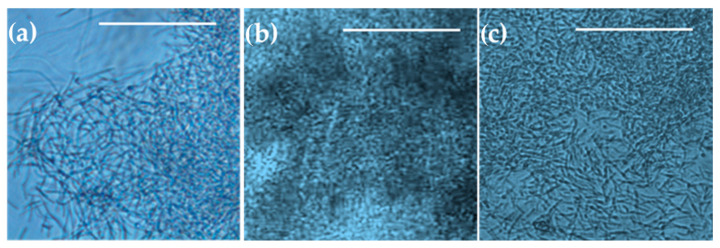
Representative images of *B. subtilis* biofilms in a microbioreactor with circular extensions taken by light microscopy after 24 h of cultivation at 36 °C in complete growth medium supplied at a flow rate of 1 μL/min and providing (**a**) 100% oxygen at a flow rate of 2 mL/min, (**b**) air at a flow rate of 2 mL/min, or (**c**) no aeration. The scale bars represent 50 µm.

Observations of *B. subtilis* biofilms cultivated in a microbioreactor with circular extensions under varying aeration conditions are presented in Figure 10. Consistent with the macroscopic observations (Figure 6a–f, Figure 7a–f and Figure 8a–f), microscopic examination revealed that the biofilms with the highest bacterial density formed under aerating conditions (Figure 10b). Biofilms formed at a 100% oxygen supply (Figure 10a) or without aeration (Figure 10c) appeared thinner or less dense.

## 4. Conclusions

In this study, a simple and cost-effective approach for the design and fabrication of microreactors with different geometries was developed. The adaptability allows rapid customization of the system to study the effects of different channel geometries on biofilm development. Moreover, the oxygen conditions can be quickly switched from anoxic to fully oxygenated, providing a wide range of conditions for testing and monitoring biofilm formation and allowing the investigation of new experimental parameters.

The use of microfluidic devices enables the creation of high-performance systems that allow the easy investigation of cell–cells interaction, the extracellular matrix, substances dissolved in the culture medium, and mechanical forces. It is also possible to observe cell cultures in real-time. These devices can be used to create a precisely defined environment in which factors such as hydrodynamic conditions, cell interactions, and the influence of various biochemical molecules and antibacterial agents can be investigated.

A major challenge in biofilm research using microbioreactors is the integration of microfluidic biofilm culture cells with in situ analytical instruments. Such integration would enable a more thorough investigation of biofilms and their behavior, representing a significant opportunity for advancements in the field.

## Figures and Tables

**Table 1:** Increase in dissolved oxygen concentrations in the liquid phase, evaluated at room temperature and different liquid flow rates, as the average of 3 measurements described in Section 2.6, with indicated standard deviations.

**Liquid flow rate [µL/min]**	5	10
$\frac{dC_{O_{2}}}{dt}$ **[mg/(L s)]**	0.279 ± 0.012	0.285 ± 0.001

**Table 2:** Comparison of the characteristics of both microbioreactor versions, determined as described in Section 2.5.

Microreactor Growth Channel	Volume [µL]	Single Surface Area [mm^2^]
without circular extensions	17 ± 4	73 ± 1
with circular extensions	28 ± 4	112 ± 1

## Data Availability

The data are available in the University of Ljubljana Repository at the following link: https://repozitorij.uni-lj.si/IzpisGradiva.php?id=150910&lang=eng, accessed on 12 August 2024.
